# Acetylsalicylic acid as an adjuvant therapy for schizophrenia

**DOI:** 10.1186/1745-6215-7-31

**Published:** 2006-10-23

**Authors:** Wijnand Laan, Jean-Paul Selten, René S Kahn, Anne-Margriet Huisman, Cobi J Heijnen, Diederick E Grobbee, Huibert Burger

**Affiliations:** 1 Julius Center for Health Sciences and Primary Care, University Medical Center Utrecht, Utrecht, The Netherlands; 2 Rudolf Magnus Institute of Neuroscience, Department of Psychiatry, University Medical Center Utrecht, Utrecht, The Netherlands; 3 Department of Rheumatology, University Medical Center Utrecht, Utrecht, The Netherlands; 4 Department of Immunology, University Medical Center Utrecht, Utrecht, The Netherlands; 5 Department of Epidemiology, University Medical Center Groningen, Groningen, The Netherlands; 6 Department of Psychiatry, University Medical Center Groningen, Groningen, The Netherlands

## Abstract

**Background:**

Findings from both epidemiological and basic research point to the possibility that NSAIDS impede the deterioration in schizophrenia.

**Methods:**

To study the efficacy of acetylsalicylic acid we will perform a randomized placebo controlled double-blind add-on trial of 80 inpatients and outpatients with schizophrenia, schizophreniform or schizoaffective disorder. Patients will be 1:1 randomized to either 3 months 1000 mg acetylsalicylic acid per day or 3 months placebo, in addition to their regular antipsychotic treatment. All patients will receive pantoprazole treatment for gastroprotection. The outcomes of this study are 3-month change in psychotic and negative symptom severity, cognitive function, and several immunological parameters.

This trial may (1) yield a new (adjuvant) therapy for schizophrenia and (2) add to the knowledge on the pathogenesis of this major psychiatric disorder.

## Background

Despite several advances in the treatment of schizophrenia, currently available pharmacotherapy does not change the course of illness or prevent functional deterioration in a substantial number of patients. Therefore, research efforts into alternative or adjuvant treatment options are needed. In this project we will empirically investigate the effect of the anti-inflammatory drug acetylsalicylic acid as an add-on to regular antipsychotic therapy on the symptoms of schizophrenia.

There are several observations and theoretical considerations that support the hypothesis that anti-inflammatory drugs can be effective in antagonizing the process underlying the clinical deterioration in schizophrenia. In numerous epidemiological and clinical studies an inverse relationship between schizophrenia and rheumatoid arthritis has been demonstrated, i.e. these conditions rarely coexist in one person [1,2]. A possible explanation for this relationship implies that the use of anti-inflammatory drugs by patients with rheumatoid arthritis protects them against the development or progression of schizophrenia. This hypothesis is supported by the observation, made in a recent population-based study, that not only rheumatoid arthritis is far less frequent among patients with schizophrenia, but also other musculoskeletal conditions commonly treated with anti-inflammatory drugs[3]. These conditions were osteoarthritis of the knee, hip and spine, low back pain, and intervertebral disc disorders.

The hypothesis of the effectiveness of anti-inflammatory drugs in schizophrenia is also supported by more basic science findings. A prominent hypothesis concerning the pathogenesis of schizophrenia implicates dysfunction of the N-methyl-D-aspartate acid (NMDA) receptor [4]. This hypothesis suggests that in schizophrenia progressive excitotoxic neuronal cell death occurs via disinhibition of glutamatergic projections to hippocampal and cortical areas [5]. In this respect, prostaglandins may play an important role in two ways: (1) because they are intermediaries in the postsynaptic signal transduction cascade of cells with NMDA-type glutamate receptors and (2) by potentiating glutamatergic transmission by inhibiting astrocytic reuptake of glutamate. Both mechanisms can potentiate excitotoxic cell death[6]. As non-steroidal anti-inflammatory drugs (NSAIDs) inhibit prostaglandin synthesis through the inactivation of cyclooxygenase, they may be of therapeutic value in schizophrenia. This mechanism may also play a role in the recently confirmed inverse relation between long-term use of NSAIDs and the risk of Alzheimer's disease[7]. The therapeutic potential of acetylsalicylic acid is further suggested by the results of an experiment performed by Grilli et al., who reported that acetylsalicylic acid and its metabolite sodium salicylate protected against neurotoxicity elicited by the excitatory amino acid glutamate in rat neuronal cultures and hippocampal slices[8].

Also according to the immunological hypothesis of schizophrenia, intervention with acetylsalicylic acid may have favourable effects. Activation of the immune system in schizophrenic patients is evident from many studies demonstrating dysregulated pro-inflammatory cytokines like interleukin (IL)-1, IL-2, and in particular IL-6 and tumor necrosis factor (TNF)-alpha [9]. The importance of the immune system in the pathophysiology of schizophrenia is indirectly supported by the evidence that many antipsychotics can act as immunomodulators [10,11]. Recently, decreased T helper-1 (TH-1) related immune parameters were found in patients with schizophrenia[11] and a shift to TH-2 like immune reactivity in a subgroup of schizophrenic patients has been hypothesized[12]. Prostaglandin E2 is known to enhance the production of TH2 cytokines via inhibition of the production of IL-12 by antigen presenting cells such as monocytes [13]. Therefore, it is conceivable that the shift towards TH-2 cytokine production in schizophrenia will be counteracted by inhibition of prostaglandin formation by acetylsalicylic acid. Also because IL-12 enhances production of TH-1 cytokines, we expect that administration of acetylsalicylic acid will also result in more TH-1 activity (e.g. γ-interferon production) relative to TH-2 activity (e.g. IL-4 production). The resulting correction of the T-helper cell imbalance may eventually reduce the symptoms of schizophrenia. Accordingly, we postulate that the greatest effect of acetylsalicylic acid will therefore be observed in those individuals with the highest relative TH-2 reactivity, i.e. the lowest IFN-γ/IL-4 ratio. It should be noted that peripheral expression of cytokines may be a reflection of the pattern of cytokine production in the brain, where cytokines are produced by glial cells, astrocytes etc., and that, additionally, anti-inflammatory cytokines of peripheral origin may signal the brain, thereby contributing to the symptoms of schizophrenia. In order to monitor the effect of the proposed immunosuppressive treatment with acetylsalicylic acid and to get more insight in the possible role of cytokines in the clinical symptoms of schizophrenia, we intend to determine TH-1 and TH-2 cytokines as well as IL-6 (general immune activation), produced by peripheral blood cells before, during and after the treatment with acetylsalicylic acid.

Alternatively, NSAIDS may ameliorate symptoms of schizophrenia by affecting neuronal membrane phospholipids. As suggested by Horrobin a decreased incorporation of arachidonic acid and docosahexaenoic acid into membrane phospholipids combined with an increased removal of these essential fatty acids hamper normal neurodevelopment and adult neuronal functioning [14]. Each of these abnormalities may be related to an altered activity of phospholipase A_2_. As acetylsalicylic acid inhibits phospholipase A_2 _this NSAID may yield clinical improvement in schizophrenia[1].

Finally, a recent study showed cyclo-oxygenase hyperactivity in platelets of schizophrenic patients[15]. If also present in the brain this further implicates acetylsalicylic acid as a potential therapeutic agent for schizophrenia.

As cyclooxygenase-1 and cyclooxygenase-2 are both constitutively expressed in the brain [16], both the older COX-1 NSAIDS such as acetylsalicylic acid and indomethacin and the newer selective COX-2 NSAIDS such as celecoxib may theoretically impede the pathologic process in schizophrenia. We are aware of only one clinical trial that examined the potential therapeutic role of NSAIDS in schizophrenia. It demonstrated a beneficial effect of the COX-2 inhibitor celecoxib as an add-on therapy during five weeks on schizophrenia psychopathology in 50 patients[17]. We decided to study the efficacy of the non-selective classical NSAID acetylsalicylic acid because of its neuroprotective effect in rat neuronal cultures and, in view of the epidemiological inverse relation between schizophrenia and rheumatoid arthritis, because of its past widespread use in the treatment of rheumatoid arthritis.

### Research questions

Does 1000 milligrams of acetylsalicylic acid daily reduce symptoms of schizophrenia? Is this effect modified by initial relative TH-2 reactivity?

### Study objectives

To determine the effect of three months additional treatment with acetylsalicylic acid on positive, negative and cognitive symptoms as well as immunological parameters in patients treated with antipsychotics for schizophrenia. A secondary objective is to examine whether this effect is modified by initial relative TH-2 reactivity.

## Design and methods

### General

We will perform a randomized, placebo-controlled, double-blind multicenter trial of 80 inpatients and outpatients with schizophrenia, schizophreniform or schizoaffective disorder.

### Inclusion criteria

To be included in the study one has to give written informed consent, be diagnosed with schizophrenia, schizophreniform or schizoaffective disorder according to DSM-IV, aged between 18 and 55 years old, with disease duration less than 10 years. All participants have to be clinically stable, meaning no change in dose of antipsychotic drugs 2 weeks before inclusion. At randomisation all participants need to have a score of at least 60 on the total score of the Positive and Negative Syndrome Scale (PANSS) with two scores of at least 4. For safety reasons participants are not allowed to have a contraindication for, or be hypersensitive to acetylsalicylic acid or pantoprazole, have a significant somatic illness or be pregnant. Participants are not allowed to use corticosteroids or chronically use of nonsteroidal anti-inflammatory drugs (NSAIDs) or platelet inhibitors. Persons with drugs dependencies are not allowed to participate.

### Screening

Patients will be recruited at the Department of Psychiatry of the University Medical Center Utrecht, 'Symfora Amersfoort', 'Psychiatric Center AMC/de Meren, Amsterdam', 'Spatie Apeldoorn', 'Stichting de Geestgronden', 'Adhesie Deventer', 'RIAGG Amersfoort' and the 'Delta pychiatric hospital'. If the treating responsible psychiatrist considers a patient eligible for study, his diagnosis will be confirmed according to the Diagnostic and Statistical Manual of Mental Disorders fourth edition (DSM-IV) using the Comprehensive Assessment of Symptoms and History (CASH). The PANSS will be filled out and the remaining inclusion criteria will be checked by taking medical history and performing a general physical examination. Finally, venous blood will be taken for the assessment of immunological parameters.

### Informed consent

The trial will be conducted in agreement with the principles of the Declaration of Helsinki (Edinburgh 2000). The investigator will explain the benefits and risks of participation in the study to each subject and will provide an informed consent form approved by the ethical review board of the University Medical Center Utrecht. The patient is then asked to sign the form prior to inclusion into the study. Data from this study will be treated confidentially and publication of the results of this study will be performed anonymously.

### Placebo run-in and adherence

A placebo run-in procedure will be set up to test the participant's adherence behavior in advance of the actual study. Patients meeting the inclusion criteria will be asked to take the add-on study medication (only placebo, no pantoprazole) during two weeks and will be asked to return the empty blisters to the researcher for administrative reasons. Only participants showing greater than 80% adherence (more than 80% of medication taken) will be randomized. During the trial the participants and their close relatives/friends will be informed about the importance of continuation of taking the study medication and returning the empty blisters to the researcher. Adherence will be checked by counting the returned empty blisters and determining plasma salicylic acid at months two and three. All participants will be kindly asked not to use any drugs such as marijuana or hashish within 24 hours before visit.

### Baseline assessments

As patients will be randomized in strata of relative TH-2 reactivity a blood sample will be taken at the beginning of the placebo run-in in order to have the immunological parameters determined by the time of randomization. At most one week before randomization, the severity of baseline psychotic symptoms will be assessed using the PANSS. In addition, a short cognitive test battery focusing on verbal learning and motor performance (e.g. California verbal learning test, Purdue Peg Board test, trail making test, HQ-Continues Performance test) will be administered.

### Randomized intervention

Patients will be randomized in a 1:1 ratio to either supplementation of acetylsalicylic acid or placebo in addition to their current antipsychotic treatment. Randomization will be performed in strata of psychiatric center (tertiary or non-tertiary) and relative TH-2 reactivity (high/low), delineated by the median IFN-γ/IL-4 ratio. To this end a computer-generated list will be produced with allocation codes in random order, balanced in the four strata by using permuted blocks. The aspirin dose is based on a trade-off between a dose that has a relatively low risk of gastro-intestinal toxicity in young persons, and the dose that is presumed to be anti-inflammatory, the latter being adopted from therapeutic doses used in the treatment of musculo-skeletal disorder[18]. Placebo will be identically packaged, looking and tasting tablets. For optimal gastro protection, all patients (also those randomized to placebo) will be given 40 milligrams of pantoprazole daily[19].

### Concomitant medication

As an analgesic the patients will be emphatically advised to take acetaminophen instead of acetylsalicylic acid or other NSAIDS. A record of all medication taken will be kept during the entire trial.

### Adverse reactions

Acetylsalicylic acid use may cause dyspeptic complaints, ulcer disease and gastrointestinal bleeding. However, due to its strong association with high age (odds ratio 1.04 per year[20]), we expect that the risk of ulcer or serious bleeding is considerably lower than 1% per year that was observed in persons over 60 years of age taking similar doses of aspirin[21]. Further, all patients will be given 40 milligrams pantoprazole daily which has shown to reduce the risk of NSAID induced gastrointestinal problems considerably[19]. Nevertheless, at every follow-up visit the participants will be asked about epistaxis, hematemesis, melena, rectal bleeding and hematuria. Further, dyspeptic complaints will be systematically recorded at every visit using an 8-item self-administered questionnaire [22]. Patients will be urged to stop taking study medication one week in advance of molar extractions or similar surgical procedures and to continue not before one week after the procedure. In case of medical emergencies the initial care will be managed by the general practitioner as in usual care, if possible after consultation of the investigator, or the person taking medical responsibility for the participants in this study (Prof. dr R.S. Kahn). At a later stage the investigator will consult the treating psychiatrist and the patient's general practitioner whether continuation in the trial is reliable.

### Study outcomes

The primary outcome of this trial is the 3-month change in positive and negative symptoms on the total PANSS score. Secondary outcomes are the 3-month change in the PANSS subscales, cognitive symptoms, immunological parameters (γ-interferon, IL-4, IL-6 and IL-12), and psychoactive medication taken during the trial.

### Follow-up assessments

At one, two, and three months, and in case of withdrawal from the study, the severity of positive and negative symptoms will be reassessed using the PANSS. The cognitive tests will be repeated at three months or at study discontinuation. Blood samples will be taken at 2 and 3 months, or at withdrawal, for determination of the Aspirin levels. All assessments will be performed blind to medication status. The overview of the visits can be seen in table 1.

**Table 1:** Overview of assessments/interventions

	Start of run-in	Baseline	1 Month	2 Months	3 Months (@)
Informed Consent	X	-	-	-	-
Screening	X	-	-	-	-
Pregnancy test	X	-	-	-	-
Randomization	-	X	-	-	-
Immunological parameters	X	-	-	X	X
Blood for DNA isolation	X	-	-	-	-
Aspirin concentration	-	-	-	X	X
PANSS	-	X	X	X	X
CASH (+)	X	-	-	-	-
Cognitive tests	-	X	-	-	X
Adverse events	-	-	X	X	X
Co-medication	X	X	X	X	X
Compliance	-	X	X	X	X

@ or withdrawal

+ if previous CASH is longer than 6 months ago

### Immunological measurements

To analyze the effect of the treatment on cytokine production, we will determine the in vitro capacity of peripheral blood mononuclear cells to produce cytokines. Heparinized blood (15 ml) will be drawn before, after two and after three months of treatment, or at earlier withdrawal. Peripheral blood mononuclear cells will be isolated by centrifugation of Ficoll isopaque. T cell cytokine production will be stimulated by incubation of cells with anti-CD28 and anti-CD2 monoclonal antibodies and supernatants will be collected after 48 hours of culture. In addition, adherent cells (monocytes/macrophages) will be stimulated for 24 hours with lipopolysaccharide to induce IL-6 and IL-12 production. Cytokine levels in culture supernatants will be analyzed by ELISA. In vivo cytokine production will be analyzed by determining cytokine levels in plasma samples obtained at the same time points. In addition, blood will be stored for future research possibilities on the immunological or genetic aspects of schizophrenia, schizoaffective and schizophreniform disorders, after permission of the patients (in the informed consent form).

### Withdrawal from the study

A patient must be withdrawn from the study when judged necessary by the responsible psychiatrist or when the patient withdraws his/her informed consent. In these instances all outcomes should be assessed.

### Study size

The planned number of patients to be included in this trial is 80, 40 in each arm. This is sufficient to show a statistically significant difference between the intervention arms of effect size (Cohen's *d*) ≥ 0.66 for the change in total PANSS score from baseline to last follow-up. It is based on an alpha of 0.05, a power (1-beta) of 0.8 and a two-sided unpaired t-test. Further, we accounted for 10% withdrawal. It should be noted that the power of the repeated measures analyses is considerably higher.

### Data analysis

The statistical significance of the difference in the change in total PANSS score, its subscores, cognitive functions, immunological parameters from baseline to last follow-up between the arms and psychoactive medication will be tested using a two-sided unpaired t-test and its magnitude will be supplied with a 95% confidence interval. In addition, all outcomes will be analyzed as dependent variables in repeated measurements analyses with treatment arm as the independent variable. To study whether the potentially beneficial effect of acetylsalicylic acid is modified by initial relative TH-2 reactivity, we will repeat the analyses in strata of IFN-γ/IL-4 ratio The analyses will primarily be 'intention-to-treat'. In addition, a per protocol analysis will be performed on those who showed more than 90% adherence. If despite randomization important baseline differences in prognostic factors exist, adjustments will be made by including the corresponding variables as independents in the multivariable models. Finally, the change in psychopathological symptoms will be related to the change in immunological parameters using multivariable regression models. In these analyses adjustments for confounders will be made when appropriate.

### Duration

We estimate that recruitment of patients will take 1 year. Follow-up will last 3 months. Assuming that data analysis and reporting require around 9 months, the total duration of the trial will be approximately 2 years.

## Competing interests

The author(s) declare that they have no competing interests.

## Authors' contributions

JPS, RSK, AMH, CJH, DEG and HB formed the original study team that developed the research question. HB wrote the study protocol, obtained local ethics approval, and obtained grant funding. WL included the participating centres and performed the study.
